# Distribution and evidence of co-infection of the two microsporidian parasites *Astathelohania contejeani* and *Nosema austropotamobii* in *Austropotamobius pallipes* complex in Northern and Central Italy

**DOI:** 10.1017/S0031182024001525

**Published:** 2024-12

**Authors:** Gianluca Fea, Daniela Ghia, Andrea Basso, Valentina Paolini, Roberto Sacchi, Tobia Pretto

**Affiliations:** 1 Dipartimento di Scienze della Terra e dell'Ambiente, Università degli Studi di Pavia, Pavia, Italy; 2 Institute of Agricultural and Environmental Sciences, Estonian University of Life Sciences, Tartu, Estonia; 3 Istituto Zooprofilattico Sperimentale delle Venezie, Centro Specialistico Ittico, Legnaro (Padova), Italy

**Keywords:** detection probability, parasite, porcelain disease, white-clawed crayfish

## Abstract

*Austropotamobius pallipes* complex is an endangered freshwater crayfish species in Europe and the assessment of the health status of its wild populations is essential for conservation purposes. The two microsporidia *Astathelohania contejeani* and *Nosema austropotamobii* have been reported to cause in *A. pallipes* complex a chronic parasitic infection, known as ‘porcelain disease’, which reduces population fitness and leads the host to death. Due to the similar macroscopic signs produced, molecular biology analyses are required to unambiguously distinguish between these microsporidia. Focusing on *A. pallipes* complex populations located in Northern and Central Italy, the present study provides an evaluation of prevalence and distribution of *A. contejeani* and *N. austropotamobii*, and investigates the variables affecting the probability of detecting infected specimens during a survey (e.g. sex, crayfish density, longitude). Microsporidia were identified in 12 populations among the 42 monitored from 2011 to 2017, with an average prevalence of 3.12% for *A. contejeani* and 3.60% for *N. austropotamobii*, the latter being reported in a wider area than previously documented (from Lombardy to Liguria Regions). Notably, crayfish co-infected by both microsporidia were also detected in 4 populations. Moreover, it was observed that the probability of detecting a crayfish with a microsporidian infection significantly increased eastwards in the studied area, especially for *N. austropotamobii*. Our distribution map for microsporidiosis, combined with molecular screening, will be useful for planning breeding and translocation efforts for *A. pallipes* complex populations.

## Graphical abstract

**Figure:**
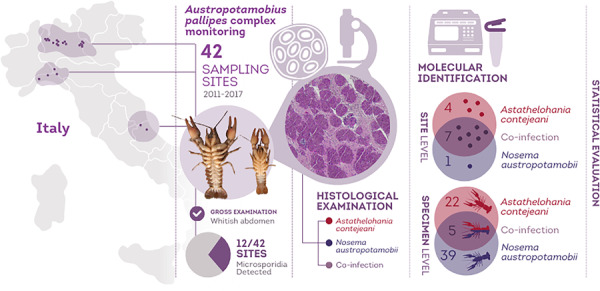

## Introduction

Crayfish are considered one of the most threatened groups of animals in freshwater environments (Richman *et al*., 2015; Manenti *et al*., 2019). Their decline can be attributed to multiple reasons, such as the expansion of human settlements, which causes habitat modification and water pollution, climate change, the invasion of alien species and the spread of infectious diseases (Zaccara *et al*., 2004; Chucholl, 2013). Infectious agents, such as bacteria, viruses, protists and fungi (Longshaw, 2011), are described as cause of mortalities in European crayfish species. The oomycete *Aphanomyces astaci*, the etiological agent of crayfish plague, is arguably one of the deadliest parasites for freshwater crayfish and is included in the worst invasive species database (DAISIE, 2009; Lowe *et al*., 2000). However, many other parasites may determine sub-lethal effects, hindering the crayfish normal activities without leading them to sudden death. In addition, there is increasing evidence that parasite-mediated effects could have a significant impact on host population dynamics, altering interspecific competition and ultimately reducing population fitness (Hudson *et al*., 2006; Haddaway *et al*., 2012; Anderson and Sukhdeo, 2013). These interactions have immediate consequences for management and conservation projects involving protected species. Therefore, according to IUCN/SSC protocols (2013), the health status of the endangered species should be considered when planning conservation and translocation actions for their populations.

The European white-clawed crayfish, *Austropotamobius pallipes* (Lereboullet, 1858) species complex (Souty-Grosset *et al*., 2006) is among the most endangered crayfish species in Europe (Kozák *et al*., 2011; Mazza *et al*., 2011). Consequently, appropriate conservation actions and policies have become of primary importance for this species and several initiatives have been implemented during the last decades all over Europe to reverse the biodiversity loss (projects financed by regional, national and European funds). Over the years, biodiversity protection plans have been performed with two main purposes, i.e. habitat restoration, where the species are still present, and reproduction in controlled environment to recover locally extinct populations. In both cases, a deep knowledge of the health condition of populations as well as the causes of local extinctions is essential to plan any conservation project (Schulz *et al*., 2002; Souty-Grosset and Reynolds, 2010). Rearing and translocation efforts of *A. pallipes* complex must be carried out following the assessment of the health status of the donor populations. Essentially, stocks must be free from crayfish plague and the specimens affected by parasites should be less than 10% (Souty-Grosset and Reynolds, 2010).

Troublesome pathogens for European freshwater crayfish are microsporidia: obligate intracellular eukaryotic parasites that are widespread over the world and are specialized in infecting almost all animals and plants (Momot and Gall, 1971; Vossbrinck *et al*., 1987; Vávra and Lukeš, 2013; Bojko *et al*., 2022). Although these parasites are lethal or sub-lethal for the hosts, some of them can be used in biological control, for example manipulating the sexual development of target arthropods (Freeman *et al*., 2010). Several genera of microsporidia are known to affect the superfamily Astacoidea (Azevedo, 1987; Longshaw, 2011; Quiles *et al*., 2019; Bojko *et al*., 2020*a*, 2020*b*; Anderson *et al*., 2021; Stratton *et al*., 2022*a*, 2022*b*, 2023*a*, 2023*b*), in particular, two species have been observed to specifically infect white-clawed crayfish (Lom *et al*., 2001; Pretto *et al*., 2018). Recently, these species were independently subjected to a taxonomic revision, primarily based on the analysis of the small subunit of the ribosomal RNA gene (SSU rRNA) (Tokarev *et al*., 2020; Stratton *et al*., 2022*b*). The two microsporidia, formerly *Thelohania contejeani* Henneguy, 1892 and *Vairimorpha austropotamobii* Pretto, 2018, were moved to other genera to avoid polyphyly of their clades, as already highlighted in previous works (Moodie *et al*., 2003; Vossbrinck and Debrunner-Vossbrinck, 2005; Stentiford *et al*., 2013). *Thelohania contejeani* was assigned to a newly defined genus becoming *Astathelohania (Thelohania) contejeani* (Stratton *et al*., 2022*b*), while *V. austropotamobii* was moved to the genus *Nosema* becoming *Nosema (Vairimorpha) austropotamobii* (Tokarev *et al*., 2020). The present paper adheres to this reassignment. As observed by Pretto *et al*. (2018), these two different microsporidia cause similar muscular lesions in white-clawed crayfish: the disease manifests itself with a noticeable whitening of the skeletal muscle as the parasite infects muscle fibres (hence the name ‘porcelain disease’), limiting movement and eventually leading the host to death (Oidtmann *et al*., 1996). However, the tropism of *N. austropotamobii* is restricted to the skeletal muscles and this parasite does not affect heart, gut musculature and nerve cord as *A. contejeani* does (Pretto *et al*., 2018). Considering the similar macroscopic signs, it is reasonable to assume that, before the work of Pretto *et al*. (2018), both parasites were improperly identified as a single species, especially when the samples were partitioned for various purposes (i.e. cytological, histopathological, molecular assays).

The survival rate of individuals affected by microsporidiosis is partly unknown, but the hosts may take months or even years to die (Oidtmann *et al*., 1996). Typically, the prevalence in white-clawed crayfish populations ranges from 0.2 to 10% (O'Keeffe and Reynolds, 1983; Mori and Salvidio, 2000), although higher rates have been reported (O'Keeffe and Reynolds, 1983; Skurdal *et al*., 1990; Dunn *et al*., 2009). *Nosema austropotamobii* has been observed only in *A. pallipes* complex in Northern Italy, but *A. contejeani* has been detected in all European indigenous crayfish (Edgerton *et al*., 2002; Dunn *et al*., 2009; Pretto *et al*., 2018). Nevertheless, the transmission of these microsporidia to new hosts remains poorly understood. Some transmission routes have been suggested (e.g. direct cannibalism of infected crayfish tissues and exposure to water contaminated with spores) developing laboratory experiments to investigate the horizontal transmission of *A. contejeani* in *A. pallipes* complex and in the invasive signal crayfish *Pacifastacus leniusculus* (Imhoff *et al*., 2012). Moreover, despite some works assessing the regional presence of *A. contejeani* in watercourses of the Liguria, Lombardy and Veneto regions (Mori and Salvidio, 2000; Quaglio *et al*., 2011; Pretto *et al*., 2018), in Italy the geographical distribution of this parasite remains largely unknown and no other studies about distribution and transmission of *N. austropotamobii* have been performed since the work of Pretto *et al*. (2018).

The present study aims to investigate the prevalence and distribution of *A. contejeani* and *N. austropotamobii*, discriminated by molecular assays, in white-clawed crayfish populations from Northern and Central Italy. In addition, this work analyses the probability of detecting specimens infected by microsporidia, testing which variables between sex, number of crayfish sampled, geographic distribution or other factors influence the probability of collecting an infected crayfish during a survey. The results are reported for the first time separately for *A. contejeani* and *N. austropotamobii*.

## Materials and methods

### Crayfish sampling

**Figure 1.**
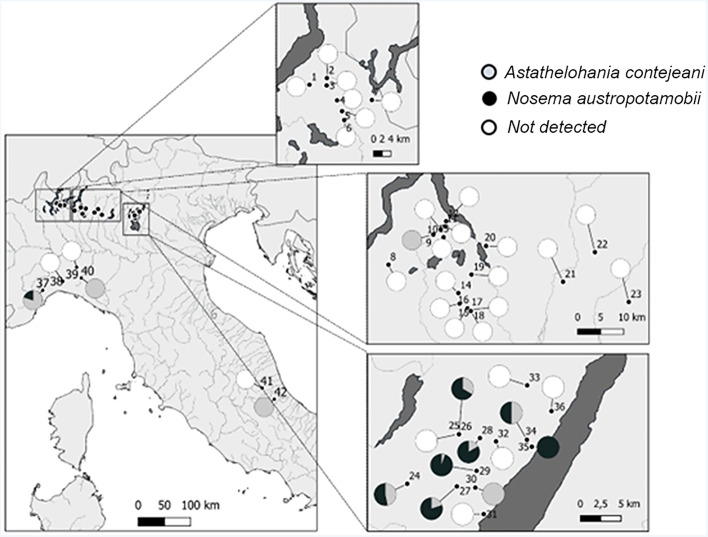
Distribution of microsporidia, detected by molecular assays, over the evaluated areas. Pies indicate the surveyed populations: grey sections indicate the presence of *Astathelohania contejeani* and black sections report the presence of *Nosema austropotamobii*. White pies represent white-clawed populations where microsporidia were not detected. Numbers refer to the watercourses-ID according to Table 1.

**Figure 2.**
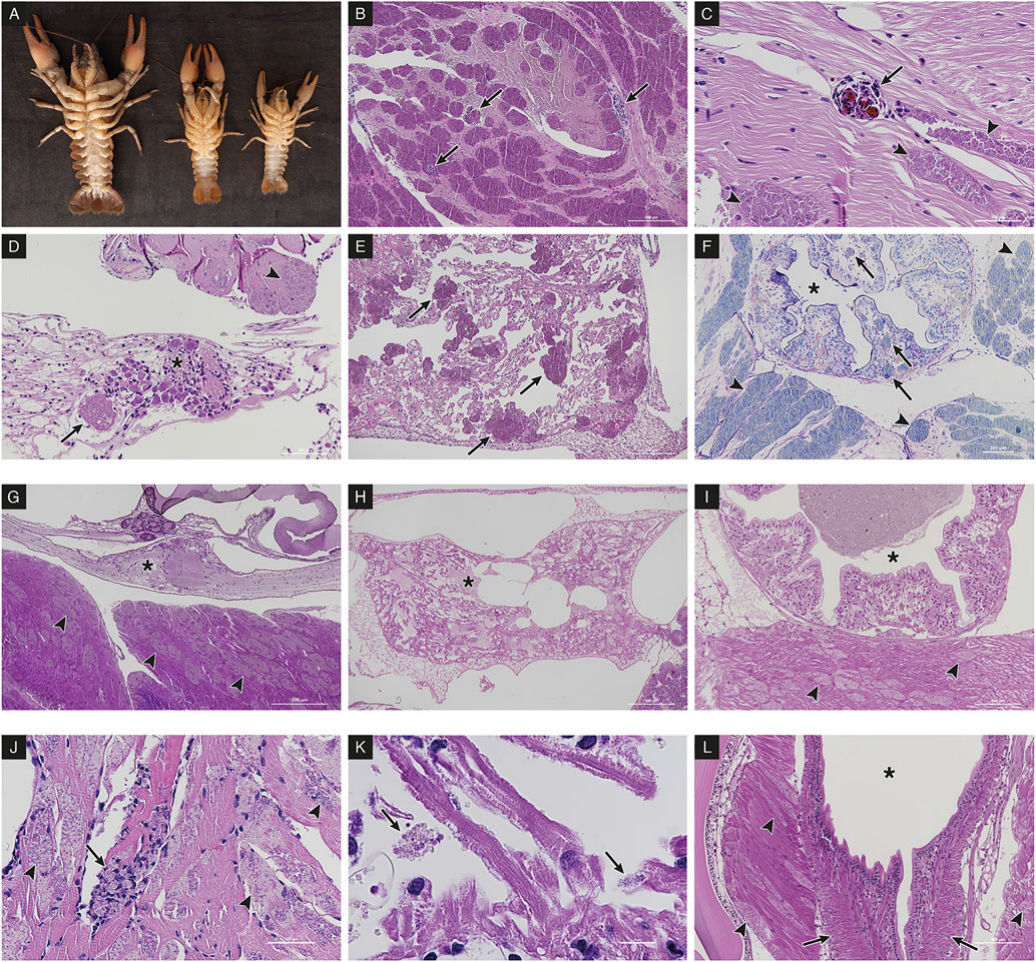
Macroscopic and microscopic detection of microsporidiosis in *A. pallipes* complex. (A) macroscopic appearance of microsporidian infection (porcelain disease) in heavily affected *A. pallipes* complex, whitish and opaque ventral skeletal musculature of the pleon and whitish discoloration of the chelipeds. (B–F) *A. contejeani* infection. (B) microscopic appearance of heavily affected skeletal muscle fibres with proliferation of *A. contejeani* and inflammatory reaction with haemocytic infiltration in muscle fibres (arrows), H-E 4×. (C) haemocytic infiltration with melanisation of affected muscle fibres (arrows) surrounded by other *A. contejeani*-infected fibres without inflammatory reaction (arrowheads), H-E 20×. (D) *A. contejeani* infection of the ventral nerve cord, microsporidian spores (arrow) between the pyrenophores of the ganglion (∗), affected skeletal muscle fibres (arrowhead), H-E, 10×. (E**)** *A. contejeani* infection of the cardiac musculature, affected cardiac muscle fibres (arrows), H-E 4×. (F) *A. contejeani* infection of hind gut (∗); the intestinal musculature appears affected (arrows), the surrounding skeletal musculature of the abdomen is also affected (arrowheads), Giemsa 4×. (G–I) *N. austropotamobii* infection. (G) *Nosema austropotamobii* severe infection of abdominal skeletal muscle fibres (arrowheads), nerve cord ganglion (∗) not affected, H-E 4×. (H) unaffected cardiac muscle fibres of *N. austropotamobii* affected specimen, cardiac lumen (∗), H-E 4×. (I) unaffected intestinal muscle fibres of *N. austropotamobii* affected specimen, infected skeletal muscle fibres (arrowheads), hind gut lumen (∗), H-E 10×. (J–L) *N. austropotamobii* – *A. contejani* co-infection. (J) Inflammatory reaction with haemocytic infiltration (arrow) in skeletal muscle fibres of co-infected *N. austropotamobii* – *A. contejeani* specimen, affected fibres with no inflammation (arrowheads) H-E 20×. (K) limited amount of microsporidian spores (arrows) in cardiac muscle fibres of co-infected *N. austropotamobii* – *A. contejeani* specimen, H-E 60×. (L) unaffected intestinal muscle fibres of co-infected *N. austropotamobii – A. contejeani* specimen, infected skeletal muscle fibres (arrowheads), hind gut lumen (∗), longitudinal section H-E 10×.

During summer and early autumn from 2011 to 2017, 42 populations of white-clawed crayfish were monitored in selected areas of the Alps and Apennines, from Northern and Central Italy (Fig. 1). Overall, 81 surveys were performed and each population was sampled on average 1.9 times (range: 1–6). 3997 freshwater crayfish were collected by hand during the night according to Peay (2004) and they were sexed scoring 1758 males and 2239 females. Then specimens were assessed for macroscopic evidence of microsporidia infection: lethargy and white appearance of the abdominal musculature and ventral surface of chelipeds. Therefore, the pleon of each specimen was evaluated recording changes from the normal greyish-translucent colour to a more whitish and opaque appearance (Fig. 2A). This sampling approach relies on the typical clinical signs of late stage infections, potentially leading to the overlooking of positive specimens at early stages of microsporidiosis. During the samplings, 95 specimens with gross signs of porcelain disease (hereafter ‘suspected’) were collected from their habitat and transferred to the laboratory. Suspected specimens were anaesthetized on crushed ice before sectioning the cephalothorax through the cephalic ganglion. The cephalothorax and the abdomen of the same specimens were injected and fixed with Davidson's solution for 48 h and post-fixed in ethanol 70% for histological examination. Concurrently, samples of abdominal musculature were stored in absolute ethanol for molecular analyses.

**Table 1.** Summary of specimens assessed for the presence of microsporidia in the surveyed watercourses

Watercourses (ID and name)		No. of evaluated specimens	Males	Females	Suspected specimens	Specimens negative to microsporidia	Specimens positive to *A. contejeani*	Specimens positive to *N. austropotamobii*	Specimens with co-presence of microsporidia
1	Marianne	11	9	2	0	–	–	–	–
2	Casarivo	95	38	57	0	–	–	–	–
3	Cantevrio	69	27	42	0	–	–	–	–
4	Valmolina	15	8	7	0	–	–	–	–
5	Des	341	142	199	0	–	–	–	–
6	Boscaccia	79	48	31	0	–	–	–	–
7	Ponticelli	32	6	26	0	–	–	–	–
8	Cosia	27	13	14	0	–	–	–	–
9	Foce	102	24	78	4	0	4	0	0
10	Cavalletto	116	43	73	0	–	–	–	–
11	Candalino	109	48	61	0	–	–	–	–
12	Posall	51	17	34	0	–	–	–	–
13	Ravella	9	4	5	0	–	–	–	–
14	Roncaria	13	4	9	0	–	–	–	–
15	Molgoretta	84	42	42	0	–	–	–	–
16	Mocur 17	98	51	47	0	–	–	–	–
17	Mocur 12	40	11	29	0	–	–	–	–
18	Rio Freddo	158	78	80	0	–	–	–	–
19	Gandaloglio	93	33	60	0	–	–	–	–
20	Ibraula	29	13	16	1	1	0	0	0
22	Ambriola	28	4	24	0	–	–	–	–
23	Predina	31	16	15	0	–	–	–	–
24	Agna	172	79	93	22	8	6	7	1
25	Lanech	7	4	3	0	–	–	–	–
26	Toscolano	111	55	56	7	0	1	2	0
27	Campiglio	125	47	78	12	2	2	8	0
28	Droanello	677	322	355	13	2	0	4	1
29	Vincerino	224	89	135	21	2	1	16	2
30	Carriole	10	5	5	1	0	1	0	0
31	Cecina	10	–	10	0	–	–	–	–
31	Giongo	258	128	130	0	–	–	–	–
32	Costa	55	37	18	0	–	–	–	–
33	San Michele	11	3	8	0	–	–	–	–
34	Aer	121	40	81	2	0	1	1	0
35	Piovere	18	5	13	1	0	0	1	0
36	Torbole	66	29	37	0	–	–	–	–
37	Valla^a^	130	55	75	6	1	3	0	1
38	Lemme^a^	33	17	16	1	1	0	0	0
39	Reponte^a^	31	16	15	0	–	–	–	–
40	Boreca^a^	30	10	20	1	0	1	0	0
41	Cesa^a^	239	117	122	1	0	0	0	0
42	Fino^a^	39	21	18	2	0	2	0	0
**Total specimens**		**3997**	**1758**	**2239**	**95**	**17**	**22**	**39**	**5**

The number of males and females, suspected specimens, and presence of different microsporidian species confirmed by molecular assays were reported. Watercourses with co-infected specimens are indicated in blue.

a Indicates watercourses located in the Apennines and thus excluded from the statistical analyses.

–, denotes absence of sampled specimens..

### Molecular analyses

After drying the excess of ethanol, total genomic DNA was extracted from up to 30 mg of the abdominal muscle tissue of 95 crayfish using a commercial kit (QIAamp DNA Mini Kit-QIAGEN). DNA was eluted with the provided buffer and quantified through NanoDrop Spectrophotometer (Thermo Fisher Scientific). The samples unsuitable for the downstream molecular biology assays (e.g.: low integrity, quality and/or quantity of DNA) were excluded, while the others were subjected to the molecular analyses.

To confirm the presence of microsporidia and identify the species, the small subunit of the ribosomal RNA (SSU rRNA) gene was analysed through end-point PCR and Sanger sequencing. As described in a previous study (Pretto *et al*., 2018), a nested PCR protocol was applied to detect *A. contejeani*, with the generic microsporidian outer primers V1f and 1492r and the *A. contejeani*-specific inner primers MIC1-5 and MIC3-4 (Imhoff *et al*., 2010). Although sensitive, this PCR often leads to chromatograms with overlapping peaks, affecting the results. This is probably due to intragenomic sequence variability between multiple copies of the SSU rRNA gene, as already observed in other microsporidia (Ironside, 2007; Pretto *et al*., 2018). To overcome this problem and confirm the presence of *A. contejeani*, the nested PCR was complemented with a specific end-point PCR protocol using primers F3 and B3 (El-Matbouli and Soliman, 2006), which target a different region of the SSU rRNA gene and produce clearer chromatograms. Amplification was carried out in a 20 μL reaction volume containing 0.5 U of Platinum Taq DNA Polymerase (Thermo Fisher Scientific), 1× PCR Buffer (−MgCl_2_), 1.5 mm MgCl_2_, 1 mm GeneAmp dNTPs Mix (Applied Biosystems), 0.25 μm each primer and 2 μL template DNA (up to 200 ng per reaction). The mixture was subjected to the following thermal protocol: 94°C for 2 min, 40 cycles of 45 s at 94°C, 45 s at 66°C and 45 s at 72°C, with a final step of 72°C for 5 min.

Given the absence of published species-specific molecular protocols to detect *N. austropotamobii,* the generic microsporidian primers V1f and 1492r (Weiss *et al*., 1994) were used to assess the presence of this parasite in the suspected specimens, amplifying the SSU rRNA gene by end-point PCR performed as described by Pretto *et al*. (2018) and followed by Sanger sequencing. Although generic, these primers allowed the identification of *N. austropotamobii* even in crayfish co-infected with both microspordia. In these cases, V1f and 1492r produced clear chromatograms for the former parasite, probably because of a higher affinity of these primers for *N. austropotamobii*, whereas *A. contejeani* was confirmed with the methods described above.

After amplification on Bio-Rad CFX96 Thermal Cycler (Bio-Rad Laboratories), PCR products were visualised on 1% agarose gel (Sigma-Aldrich) stained with GelRed (Biotium) and then sequenced using the BigDye Terminator v3.1 Cycle Sequencing Kit on an ABI PRISM 3130xl Genetic Analyzer (Applied Biosystems). The obtained sequences were then compared to the GenBank database using BLASTn.

### Histological examination

Histological processing of the suspected specimens was performed following standard protocols, as described in details by Pretto *et al*. (2018). Briefly, longitudinal sections of cephalothorax and abdomen were dehydrated and embedded in Paraplast^®^, 3 μm thin sections were stained alternatively with Harris's haematoxiline and eosine-floxine or Giemsa stain, mounted in Eukitt® resin and were observed with a Nikon H550L microscope at 40–1000×. Digital images were captured using a Nikon DS-Ri2 integrated camera and NIS Elements 5.30 software (Nikon, Japan). Presence of microsporidia at different developmental stages inside the skeletal musculature was recorded, with substitution of normal muscle fibrils with free spores and sporophorous vesicles. Haemocytic infiltrations or melanisation of affected skeletal muscle fibres were evaluated along with the distribution of microsporidian developmental stages in different tissues (heart, intestinal musculature, nerve cord) other than skeletal muscle. These characteristics were useful to distinguish between the two microsporidian species or to suspect a co-infection.

### Statistical analyses

The analysis of factors affecting the probability of detecting infected individuals was restricted to the populations of the Northern Italian Alps, excluding the two populations from Central Italy (with IDs 41 and 42), as well as four populations from the Apennines (with IDs 37, 38, 39, and 40) (Fig. 1). This restriction was made to ensure that the populations studied had similar watercourses with the same features. Consequently, the sample included 70 surveys on 36 populations for a total of 3495 individuals (1522 males and 1973 females). Firstly, we looked for the variables which could affect the probability of detecting a suspected crayfish during a survey. Hence, a generalized linear mixed model (GLMM) was performed with binomial error distribution including as predictors the following variables for each survey: the total number of observed individuals, the longitude (normalized UTM × coordinate), the sampling year (normalized), and the sex ratio (males to females). Population entered the model as random factor accounting for repeated sampling within site. In a second analysis, variables that could influence the probability of observing an infected specimen were evaluated. To do so, a GLMM with binomial error distribution was performed using sex, parasite (*A. contejeani vs N. austropotamobii*), time (normalized year of collection), and longitude (normalized UTM × coordinate) as predictors, while the site entered the model as random effect.

All two-way interactions with respect to parasite were also considered (i.e. parasite × sex, parasite × longitude, parasite × year) in order to evaluate differential effects on the probability to detect infected specimen according to sex, geographic gradient and time. As in the previous analysis, population entered the model as random factor accounting for among site variation in the probability of detecting infected crayfish. The initial models were simplified using backward elimination of the non-significant terms (likelihood ratio *χ*^2^ test) and residuals of the initial models were checked for normality and homoscedasticity (Karaman, 1970; Zuur *et al*., 2009). Analyses were performed using the package lme4 (Bates *et al*., 2015) in R ver. 3.6.0 (R Core Team, 2022) and, unless otherwise stated, reported values correspond to mean and standard errors.

## Results

### Macroscopic evaluation, molecular analyses and histological examination

According to macroscopic examination, 15 populations among the 42 surveyed (35.71%) had animals with gross signs of infection (Fig. 2A), while in the remaining 27 populations no suspected crayfish were recorded. A total of 95 suspected crayfish were collected (out of 2060 evaluated specimens in the suspected populations), with an overall suspect infection percentage of 4.61% and a relative intra-population rate of presumed infestation ranging from 0.42% (ID 41) to 12.79% (ID 24). Out of the crayfish collected, 12 were excluded after the extraction, while 83 were subjected to the analyses to test the presence of both microsporidia. The sequences retrieved from the obtained amplicons with primers F3-B3 (El-Matbouli and Soliman, 2006) and V1f-1492r (Weiss *et al*., 1994) were attributed to *A. contejeani or N. austropotamobii* respectively, matching with the sequences available in GenBank (MF344630 and MF344634), with values of coverage and similarity above 99.9%. The molecular analyses confirmed the presence of microsporidia in 66 specimens belonging to 12 different populations. Specifically: in 17 specimens (17.89% of the suspected crayfish) collected from 7 populations, microsporidia were not detected; while 22 specimens collected from 10 populations were infected solely by *A. contejeani* and 39 specimens from 7 populations were affected exclusively by *N. austropotamobii*. In addition, 5 crayfish from 4 sites resulted co-infected by both microsporidia (Fig. 1, Table 1). The average value of prevalence for the two microsporidia in the infected populations was 3.12% (with a maximum of 10%) and 3.60% (with a maximum of 8.04%) for *A. contejeani* and *N. austropotamobii*, respectively.

Histological evaluation corroborates the results obtained with the molecular methods. Positive specimens for *A. contejeani* showed variable inflammation with haemocytic reaction and melanisation of skeletal muscle fibres (Fig. 2B and C), and variable parasitic proliferation in heart, nerve cord and intestinal musculature of the hind gut (Fig. 2D–F). Whereas, specimens positive for *N. austropotamobii* showed diffuse presence of affected skeletal muscle fibres with no involvement of nerve cord (Fig. 2G), heart (Fig. 2H) or intestinal musculature (Fig. 2I). Moreover, no inflammatory response or melanisation was observed in affected fibres. Regarding the specimens showing co-infection by the two microsporidia *via* molecular examination, histology showed diffuse presence of affected skeletal muscle fibres with very limited inflammatory response (Fig. 2J) and mildly affected heart muscle fibres (Fig. 2K) only in three specimens (from Droanello, Valla and Vincerino populations). This histological appearance is compatible with chronic and diffuse infection by *N. austropotamobii* and limited (putative early stage) infection by *A. contejeani*. No histological evidence of the opposite infection status, such as diffuse chronic *A. contejeani* proliferation and precocious *N. austropotamobii* infection has been observed and corroborated by molecular identification: i.e. microsporidian infection of skeletal, cardiac, intestinal muscle fibres and nerve cord with inflammatory response associated with co-infection evidence by molecular methods (i.e. positive results for *A. contejeani* by specific F3 and B3 primer set and positive results for *N. austropotamobii* with generic V1f and 1492r primer set).

### Probability of detecting suspected crayfish during a survey

**Figure 3.**
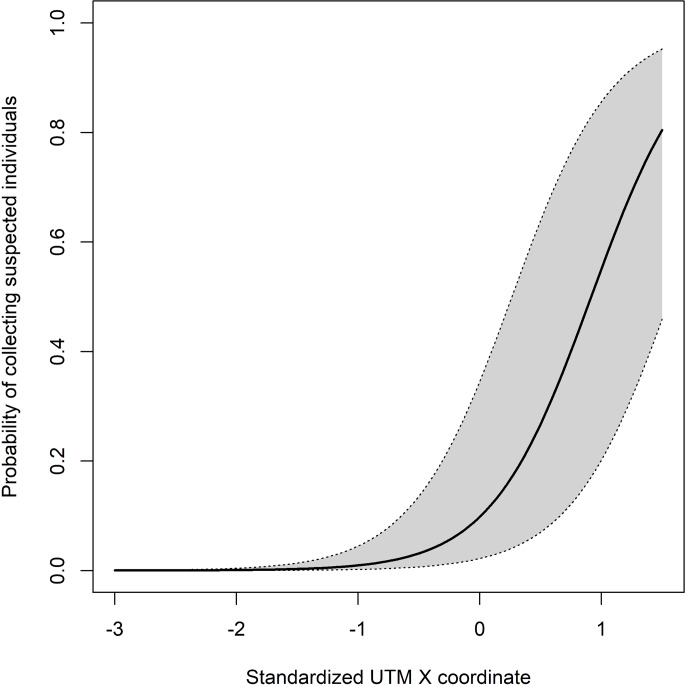
GLMM predicted probabilities of collecting suspected individuals (see Materials and Methods for details) during a single survey along a longitudinal cline. The thick line represents the predicted value for a site, whereas the grey area represents the 95% interval of variation of sites around prediction. Increasing values in the x-axis corresponds to a West-to-East cline.

The probability of finding a suspected crayfish in a single survey did not depend on the number of specimens examined (*P*_removal_ > 0.81) or on the sex ratio in the collected crayfish (*P*_removal_ > 0.82). However, the significant effects of the sampling year (LR-*χ*^2^ = 7.29, d.f. = 1, *P* < 0.0069) and the longitude (LR-*χ*^2^ = 10.89, d.f. = 1, *P* < 0.001) were detected, suggesting that the occurrence of an infected crayfish in a survey increased eastwards (Fig. 3), and decreased over the years. Nevertheless, this finding probably arose from the large number of negative outcomes obtained during 2017 (95% compared to a range of 40–75% of previous years). To control this effect, the model was ran again excluding the 20 surveys collected in 2017. This model did not support the effect of sampling year (LR-*χ*^2^ = 0.11, d.f. = 1, *P* = 0.74) but confirmed that of longitude (LR-*χ*^2^ = 15.51, d.f. = 1, *P* < 0.001).

#### Factors affecting the chance of microsporidian infection

**Figure 4.**
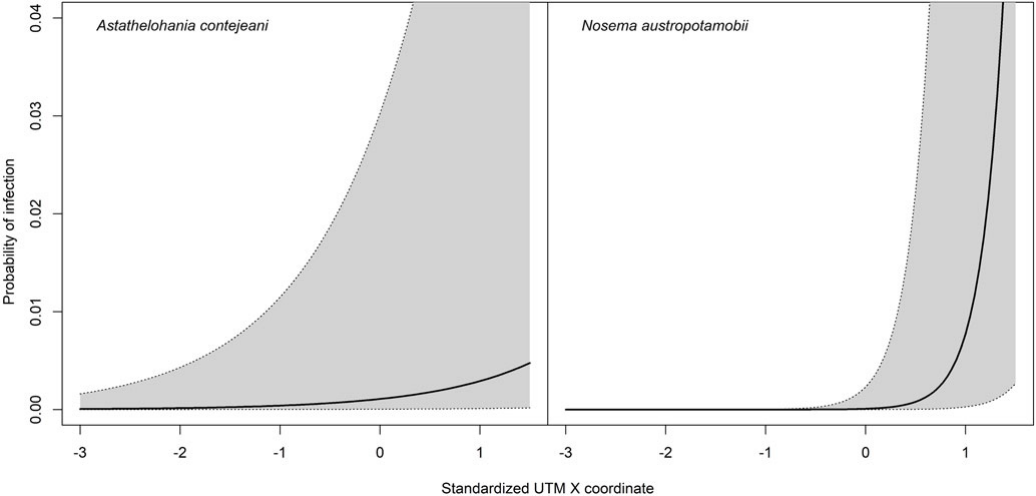
GLMM predicted probabilities of infection by *A. contejeani* (left panel) and *N. austropotamobii* (right panel) along a longitudinal cline. The thick line and grey area as in Fig. 3. Increasing values in the x-axis corresponds to a West-to-East cline.

The probability of observing a crayfish with a microsporidian infection (confirmed by molecular biology) during a survey was independent from sex (*P*_removal_ > 0.48) and sampling year (*P*_removal_ > 0.13), but was significantly affected by the parasite × longitude interaction (LR-*χ*^2^ = 12.35, d.f. = 1, *P* = 0.0004) **(×**Fig. 4). The probability significantly increased eastwards for both microsporidia, but the trend was more pronounced in the infections caused by *N. austropotamobii* (*β* = 4.57 ± 2.65) rather than by *A. contejeani* (*β* = 0.98 ± 0.56).

## Discussion

### Molecular analyses and histological examination

In this study, molecular techniques were applied to identify *Astathelohania contejeani* and *Nosema austropotamobii* in the macroscopically affected white-clawed crayfish, as the macroscopic lesions and histological appearance of the two microsporidia show notable similarities, with few diagnostic traits available to discriminate between them. These traits are limited to tissue tropism, inflammatory response, ratio of free spores and sporophorous vesicles in muscle imprints and the evaluations of ultrastructure (Pretto *et al*., 2018). Out of these, the differences between tissue tropism and immune response activation, with haemocytic diapedesis, necrosis and melanisation of affected muscle fibres, are the most useful and convenient features in discriminating between these pathogens during the histological investigation. However, the histological examination requires high expertise both in the sampling/processing phase and in the observation/description of the histological slides. This requirement is overcome by the potential of molecular diagnostic procedures, as already demonstrated for many other pathogens (Yang and Rothman, 2004; Procop, 2007; Ghosh and Weiss, 2009; Davidovich *et al*., 2022; Miswan *et al*., 2022).

Besides recording the co-infection of these two microsporidia in the same watercourses (5), as already reported by Pretto *et al*. (2018), specimens (5 crayfish from 4 sites) in which both microsporidia occurred together within the same host were also documented. Before this study, the co-infection of these two parasites in the same specimen was described only in a single crayfish collected from a population located in the Northern Apennines (Paolini *et al*., 2022) and was never observed in the prealpine and alpine areas. This interesting finding shows that the coexistence of these two microsporidia in the same specimen was not an isolated phenomenon, but is more frequent and widespread than previously reported. It is therefore relevant to explore the effects of this co-infection in white-clawed crayfish. According to the histological examinations, in all available samples showing the co-infection of the two microsporidia, the amount of *N. austropotamobii* was always predominant. A limited inflammatory response of skeletal muscle fibres was observed, while the infection of cardiac musculature was sporadically detected, but it was notably reduced compared with crayfish affected only by *A. contejeani.* Based on these observations, it is reasonable to suppose that, in case of co-infection of the two microsporidia in the same host, the tissue tropism and severity of infection usually associated with *A. contejeani* may be limited by the competition with *N. austropotamobii*, thus leading the co-infected crayfish to exhibit clinical signs, related to heart and putative nervous system impairment, intermediate between crayfish infected only with *A. contejeani* or *N. austropotamobii*. In this study, the opposite case did not occur, i.e. a predominance of *A. contejeani* and a reduced amount of *N. austropotamobii* in the same host. In case, this scenario would be characterised by extensive infection of cardiac and intestinal musculature and nervous cord, with inflammatory respose visualised by histology together with positive molecular results for both *N. austropotamobii* and *A. contejeani*. Possible reasons could be proposed (e.g. the stage of infection), but further studies are needed to clarify this particular aspect and understand how the two microsporidia compete/interact with each other in the same host: for instance, if a pre-existent infection with a microsporidium facilitates or inhibits further infections by other microsporidian species. In this regard, it would be useful to establish and apply a specific and sensitive PCR assay to test the presence of *N. austropotamobii* in the sampled crayfish.

### Prevalence, distribution and statistical evaluation

This study confirmed the presence of microsporidia by molecular analyses in 28.57% of the surveyed *A. pallipes* complex populations in the evaluated Italian areas**.** In the positive populations, prevalence of infected specimens was ≤10%, in agreement with most of the published data on microsporidiosis (Cossins and Bowler, 1974; O'Keeffe and Reynolds, 1983; Vogt, 1999; Mori and Salvidio, 2000; Hutchings *et al*., 2009). Our survey also provided relevant insights into the geographical distribution of the two microsporidia in white-clawed crayfish populations in Northern Italy, especially for *N. austropotamobii*. This species had only been reported in the eastern part of Lombardy, specifically in the Vincerino and Agna streams (Pretto *et al*., 2018), and in a single Apennine population at Lake Cerreto (Paolini *et al*., 2022). Our data confirm the presence of infected populations in prealpine streams between Iseo Lake and Garda Lake. However, *N. austropotamobii* has also been observed further west, in Liguria region (Valla, Watercourse ID 37), indicating that it is more widespread than previously documented. To understand the presence of *N. austropotamobii* in Valla stream and Cerreto Lake, far from where it was consistently detected, further analyses should involve the genetic characterization of both crayfish populations and pathogens in the surrounding areas. Notably, in the Valla stream, white-clawed crayfish coexists with the American signal crayfish *Pacifastacus leniusculus* (Ghia *et al*., 2017, 2018; Ercoli *et al*., 2021), providing the chance for further studies on the horizontal transmission of the considered microsporidia between the two crayfish species in the natural environment, which has been only investigated in an artificial setting for *A. contejeani* (Imhoff *et al*., 2012).

The prevalence of microsporidia in a population could be influenced by various factors, such as water flow or crayfish density (Cossins and Bowler, 1974; Brown and Bowler, 1977; Skurdal *et al*., 1990). In our study, the watercourses features were not included in the statistical analyses, since they are similar among the small streams in the Lombardy pre-alpine area. According to our statistical analyses, the probability of finding a suspected crayfish in a single survey did not depend on the number of individuals examined. Instead, our model detected the influence of longitude: the probability of collecting a suspected crayfish increased from West to East in the tested areas. This geographic gradient does not appear to be influenced by the sex of crayfish, in agreement with Dunn *et al*. (2009), and is independent from the number of crayfish evaluated and population density, contrary to Skurdal *et al*. (1990). The probability of detecting an infected specimen, confirmed positive by molecular biology analyses, was significantly affected only by the parasite × longitude interaction and increased eastwards, more prominently in the infections caused by *N. austropotamobii*. Our model did not allow testing the effect of parasite and longitude separately. It is supposed that the probability of detecting an infected specimen is mainly influenced by the species of microsporidia involved. Considering the higher severity of the infection usually associated with *A. contejeani*, as already discussed above, it is feasible that this parasite could lead its host to death more rapidly than *N. austropotamobii*. This would limit the number of crayfish infected by *A. contejeani* in a population, compared to *N. austropotamobii*, hence reducing the probability of detecting this parasite. Furthermore, it is necessary to consider the possible influence of the sampling method on data collection. Haddaway *et al*. (2012) reported that parasitism had a significant effect on predation, with an observed reduction in the predatory strength of *A. pallipes* (55% less mobile prey and 41% less non-mobile food), which may reflect a reduced muscle function and a lower metabolic rate in infected hosts, in accordance with the skeletal musculature infection caused by microsporidia. Similar observations were also reported by Brown and Bowler (1977) and Skurdal *et al*. (1990), who noted higher infection levels in scuba-diving sampling compared to trapping. Therefore, the capture method applied in the present study could be biased towards infected animals. Councurrently, this method does not allow the detection of crayfish at early stage of disease (i.e. without evident signs of infection), probably leading to a certain percentage of false negative populations. However, in our study the same sampling technique was consistently applied across years and watercourses (all crayfish were caught by hand during the night), avoiding the issue of multiple methods affecting data collection.

### Implications for conservation efforts of the endangered crayfish

Many biodiversity conservation projects aim to enhance the survival of endangered crayfish species through reintroduction and translocation programs (Kemp and Hiley, 2002; Kemp *et al*., 2003; Horton, 2009), with health monitoring playing an essential role in these efforts. Regarding this aspect, the macroscopic assessment of microsporidia is limited by the incubation time between infection and the appearance of clinical signs. Consequently, a quarantine period of at least six months is recommended before macroscopic evaluation (Brown and Bowler, 1977; Mazylis, 1978; Diéguez-Uribeondo *et al*., 1997), although a long quarantine is not always practical or effective.

To address these challenges, crayfish could be collected or translocated by taking advantage of a distribution map of the infected populations (such as the one presented in this paper for the *A. pallipes* complex populations located in Northern Italy), which is an additional tool that highlights the water bodies where the presence of microsporidia was already detected. Furthermore, non-lethal sampling techniques, such as environmental DNA (eDNA) and biopsy, combined with molecular screening could be used to assess populations more effectively.

To ensure statistically meaningful results despite practical constraints, WINEPI software (Abramson, 2011), suggests inspecting at least 65 crayfish per population through visual screening, assuming an average prevalence of 3% and a 95% confidence interval in populations of at least 100 specimens. Since the populations are often not so abundant, health monitoring can be divided into two sessions, inspecting around 30 specimens *per se*ssion. This approach would improve conservation efforts by identifying and removing crayfish with visible signs of late-stage infection and detecting microsporidia while balancing accuracy with practical limitations.

## Data Availability

All the data collected are presented in Table 1. However, the coordinates of the watercourses are not provided because the species is protected by the Habitats Directive. If necessary, it is possible to contact the authors.
